# Genetic Determinants for Bacterial Osteomyelitis: A Focused Systematic Review of Published Literature

**DOI:** 10.3389/fgene.2021.654792

**Published:** 2021-06-17

**Authors:** Xiaoping Xie, Jiangbi Li, Feng Gu, Ke Zhang, Zilong Su, Qiangqiang Wen, Zhenjiang Sui, Pengcheng Zhou, Tiecheng Yu

**Affiliations:** Department of Orthopedics, The First Hospital of Jilin University, Changchun, China

**Keywords:** osteomyelitis, genetic polymorphism, genotype, susceptibility, systematic review

## Abstract

**Background:** Osteomyelitis is an inflammatory process characterized by progressive bone destruction. Moreover, chronic bacterial osteomyelitis is regarded as a difficult-to-treat clinical entity due to its long-standing course and frequent infection recurrence. However, the role of genetic factors in the occurrence and development of bacterial osteomyelitis is poorly understood.

**Methods:** We performed a systematic review to assess the frequency of individual alleles and genotypes of single-nucleotide polymorphisms (SNPs) among patients with bacterial osteomyelitis and healthy people to identify whether the SNPs are associated with the risk of developing bacterial osteomyelitis. Then, gene ontology and Kyoto Encyclopedia of Gene and Genomes analyses were performed to identify the potential biological effects of these genes on the pathogenesis of bacterial osteomyelitis.

**Result:** Fourteen eligible studies containing 25 genes were analyzed. In this review, we discovered that the SNPs in *IL1B, IL6, IL4, IL10, IL12B, IL1A, IFNG, TNF, PTGS2, CTSG, vitamin D receptor* (*VDR*), *MMP1, PLAT*, and *BAX* increased the risk of bacterial osteomyelitis, whereas those in *IL1RN* and *TLR2* could protect against osteomyelitis. The bioinformatic analysis indicated that these osteomyelitis-related genes were mainly enriched in inflammatory reaction pathways, suggesting that inflammation plays a vital role in the development of bacterial osteomyelitis. Furthermore, functional notation for 25 SNPs in 17 significant genes was performed using the RegulomeDB and NCBI databases. Four SNPs (rs1143627, rs16944, rs2430561, and rs2070874) had smaller scores from regulome analysis, implying significant biological function.

**Conclusion:** We systematically summarized several SNPs linked to bacterial osteomyelitis and discovered that these gene polymorphisms could be a genetic factor for bacterial osteomyelitis. Moreover, further large-scale cohort studies are needed to enhance our comprehensive understanding of the development of osteomyelitis to provide earlier individualized preventions and interventions for patients with osteomyelitis in clinical practice.

## Introduction

Osteomyelitis is an inflammatory process characterized by progressive bone destruction. It is a bone infection mainly caused by microorganism invasion, and *Staphylococcus aureus* is the bacterial pathogen frequently isolated from patients with posttraumatic and hematogenous osteomyelitis (Lew and Waldvogel, 2004; Olson and Horswill, 2013). Commonly, according to the etiology, osteomyelitis could be divided into three types: posttraumatic osteomyelitis, hematogenous osteomyelitis, and osteomyelitis caused by vascular insufficiency (Lew and Waldvogel, 2004). Posttraumatic osteomyelitis predominantly occurs following open traumatic fracture, skeleton surgery, or prosthetic joint replacement. Meanwhile, hematogenous osteomyelitis typically occurs in children, characterized by the spread of bacteria from a lesion to the bone through the bloodstream. Osteomyelitis secondary to vascular insufficiency particularly occurs in patients with diabetes or diabetic foot infection (Lew and Waldvogel, 2004). Chronic osteomyelitis is regarded as a difficult-to-treat clinical entity due to its long-standing course and frequent infection recurrence, with a high risk of morbidity and mortality (Valour et al., 2014). Patients with chronic osteomyelitis have a higher incidence of psychosocial impairment (Tseng et al., 2014) and have healthcare and economic burden (Kapadia et al., 2016). The pathogenesis of osteomyelitis is linked to both environmental and genetic factors. Currently, several pieces of evidence have suggested that genetic predisposition plays an essential role in the pathogenesis of osteomyelitis (Chen et al., 2017; Paludo et al., 2017). With rapid development and application of sequencing and genetic association analysis for complicated diseases, genetic variants that potentially contribute to the occurrence of osteomyelitis are widely investigated.

Single-nucleotide polymorphisms (SNPs) of DNA sequences are common in the population. Many SNPs in genes related to the occurrence of osteomyelitis have been widely reported. For example, *TaqI* (rs731236) and *FokI* (rs2228570) of the vitamin D receptor (*VDR*) gene polymorphism may contribute to the susceptibility of chronic osteomyelitis (Jiang et al., 2016). This review was conducted to examine the individual allele frequency or genotype of gene variants among patients with osteomyelitis to identify whether gene polymorphisms are associated with the probability of developing osteomyelitis.

## Materials and Methods

This systematic review was performed based on the Preferred Reporting Items for Systematic Reviews and Meta-Analyses (PRISMA) guidelines (Moher et al., 2009).

### Literature Search Strategy

A systematic literature search was conducted using the PubMed, EMBASE, and Web of Science databases. The following terms— “genetic polymorphism,” “genetic variants,” “DNA polymorphism,” “single-nucleotide polymorphism,” “SNP,” “osteomyelitis,” and “bone infection” —were used to search all eligible studies on the relationship between SNPs and the risk of osteomyelitis and published until the end of December 2020. Additional studies were identified by screening the reference lists of the included studies. English articles were included in this review. Detailed search strategies are provided in Supplementary Table 1.

### Inclusion and Exclusion Criteria

The selected studies fulfilled the following inclusion criteria:

- Bacterial osteomyelitis was definitely diagnosed according to the standard criteria.
- The study reported the association between SNPs and susceptibility to osteomyelitis.
- Sufficient data could be extracted from the study.
- The study was limited to case–control or cohort studies on humans.

The exclusion criteria were as follows:

- The same study was duplicated or overlapped.
- Case reports, letters, meta-analyses, reviews, or studies on animals were excluded.
- The study reported the correlation between other types of genetic polymorphisms and osteomyelitis.
- The study reported the association of gene polymorphism with non-bacterial osteomyelitis.

We screened the literature by title and abstract. For the abstracts that we could not fully access, we obtained the full text to complete the assessment before we decided to include or exclude them. Two reviewers independently screened the articles and discussed uncertain publications to resolve disagreements.

### Quality Assessment

Two reviewers (XP.X. and JB.L.) independently conducted a quality methodological assessment of the studies included in the review based on the Newcastle–Ottawa Scale (NOS; Stang, 2010). The NOS scores ranged from zero stars to nine stars. Studies with NOS scores of below six stars were excluded in the review, and those with a score of at least six stars were considered good quality. We resolved disagreements by discussion or consultation with the third reviewer (F.G.), if necessary.

### Biological Function Annotation of Targeted Genes and SNPs

The protein–protein interaction network of the genes included in the review was constructed using the Search Tool for the Retrieval of Interacting Genes (STRING, version 11.0) database (https://string-db.org/). Hub genes are discovered using Cytoscape (version 3.4.0). Gene ontology (GO) and Kyoto Encyclopedia of Gene and Genomes (KEGG) analyses were performed to analyze the cell components, biological processes, and pathway enrichment of these osteomyelitis-related genes based on the DAVID database (version 6.8, https://david.ncifcrf.gov/tools.jsp/). *P* < 0.05 were used to denote statistical significance, and ≥3 enriched genes were considered significant as well.

The potential biological functions of SNPs were evaluated using the RegulomeDB (version 2.0, https://www.regulomedb.org/regulome-search/) and NCBI (https://www.ncbi.nlm.nih.gov/) databases. The two databases could predict whether gene variants affect transcription factor binding and gene expression.

### Data Extraction and Statistical Analysis

The data extracted from each included studies were as follows: first author, year of publication, nation, study design, number of cases and controls, name of gene SNP, distribution of genotype and allele frequency in cases and controls, genotype method, and Hardy–Weinberg equilibrium (HEW) in controls. Two reviewers independently extracted important data from each included study. The data from each study were as follows: odds ratios (ORs), 95% confidence interval (CI), *p*-value, and genetic model. *P* < 0.05 were used to denote statistical significance.

## Results

### Characteristics of the Included Studies

**Figure 1:**
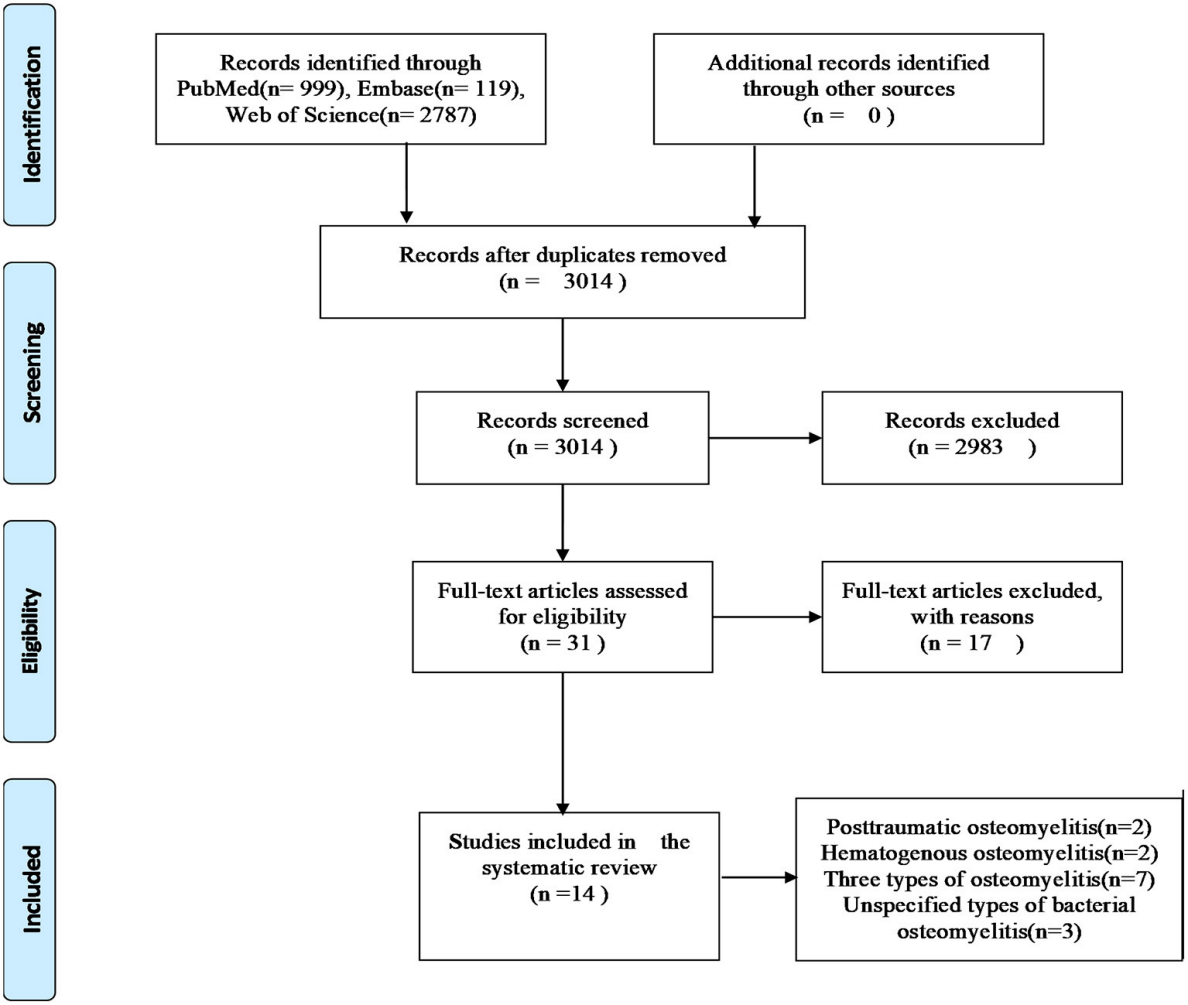
The detailed process of document selection.

Based on our literature search strategies, 3,905 publications were obtained from three databases (Supplementary Table 1), of which 891 were removed because of duplication. Then, 2,983 of the remaining publications were excluded by browsing their titles and abstracts. The full texts of 31 publications were obtained, of which 17 were excluded because of the following reasons: nine studies focused on chronic non-bacterial osteomyelitis; in two studies, SNPs were not involved; two studies were not case–control studies; in three studies, controls were not healthy individuals; and one study had inconsistent data. Eventually, 14 studies fulfilled our inclusive criteria (including 1,248 cases and 1,712 controls). The document selection process is detailed in the flowchart (Moher et al., 2009), shown in Figure 1. Among the 14 included studies, two have reported the associations between SNPs and posttraumatic bacterial osteomyelitis (Wang et al., 2017; Jiang et al., 2020), and two studies have reported the relationship between SNPs and hematogenous bacterial osteomyelitis (Osman et al., 2015, 2016). Seven studies have reported the associations between SNPs and three types of osteomyelitis (posttraumatic, hematogenous, and vascular insufficiency-related bacterial osteomyelitis; Asensi et al., 2003; Montes et al., 2006; Valle-Garay et al., 2013; Jiang et al., 2016; Hou et al., 2018; Perez-Is et al., 2019; Zhao et al., 2020). The other three studies have reported the relationship of SNP with unspecified types of bacterial osteomyelitis (Ocana et al., 2007; Tsezou et al., 2008; Kong et al., 2017). This systematic review involved 1,248 patients with bacterial osteomyelitis, including 719 patients with posttraumatic bacterial osteomyelitis, 190 patients with hematogenous bacterial osteomyelitis, 98 patients with vascular insufficiency-related bacterial osteomyelitis, and 241 patients with unspecified types of bacterial osteomyelitis. The characteristics of selected studies are summarized in Table 1. The qualities of the included studies were evaluated using the NOS, and the scores are also presented in Table 1. It should be noted that among the five articles from the same authors, the cases and controls of each article shared their own data. The two articles in the team of Asensi et al. also shared their own data (Asensi et al., 2003; Perez-Is et al., 2019). All genotyped gene SNPs in 11 studies complied with the Hardy–Weinberg equilibrium (HWE) for healthy controls (*p* > 0.05), and three studies did not mention the HWE results.

**Table 1:** Characteristics of included studies.

**References**	**Nation**	**SNPs**	**Case**	**Control**	**Types of case and control**	**Study design**	**Genotype method**	**Nos**.
Zhao et al. (2020)	China	**IFN-γ:** rs2430561(874T/A)	233 (185/48)	200 (147/53)	Patients (P/H/V^*^:189/23/21) vs. healthy individuals	Case–control study	SNaPshot system	7
Perez-Is et al. (2019)	Spain	**CTSG:** rs45567233	329 (245/84)	415 (295/120)	Patients (P/H/V:196/56/77) vs. healthy individuals	Case–control study	PCR-RFLP	7
Hou et al. (2018)	China	**TNF-α:** rs1799964, rs1800630, rs1799724, rs1800750,rs1800629, rs361525	233 (185/48)	200 (147/53)	Patients (P/H/V:189/23/21) vs. healthy individuals	Case–control study	SNaPshot system	7
Jiang et al. (2016)	China	**VDR:** rs731236, rs1544410, rs2228570, rs7975232	233 (185/48)	200(147/53)	Patients (P/H/V:189/23/21) vs. healthy individuals	Case–control study	SNaPshot system	7
Valle-Garay et al. (2013)	Spain	**tPA:** rs4646972, rs1799889	261 (186/75)	299 (205/94)	Patients (P/H: 224 /37) vs. healthy individuals	Case–control study	PCR-RFLP	8
Montes et al. (2006)	Spain	**TLR2:** Arg753Gln **TLR4:** rs498670, rs498671	80 (54/26)	155	Patients (P/H:67/13) vs. healthy individuals	Case–control study	PCR-RFLP	8
Asensi et al. (2003)	Spain	**IL-1α:** rs1800587 **IL-1β:** rs1143634 **TNF-α:** rs 1800629 **IL-6:** rs1800795	52	109	Patients (P/H:43/9) vs. healthy individuals	Case–control study	PCR-RFLP	8
Wang et al. (2017)	China	**COX-2:** rs20417, rs689466	189 (156/33)	220 (167/53)	Patients (P:189) vs. healthy individuals	Case–control study	SNaPshot system	7
Jiang et al. (2020)	China	**IL-1α:** rs17561, rs1800587 **IL-1β:** rs16944, rs1143627, rs1143634, rs2853550 **IL1RN:** rs4251961, rs419598, rs315951 **IL-4:** rs2243248, rs2243250 **IL-6:** rs1800795, rs1800796, rs1800797 **IL-8:** rs4073, rs2227306, rs2227307 **IL-10:** rs3024491, rs3024496, rs1800871, rs1800872, rs1800896 **IL17A:** rs2275913 **IF17F:** rs763780	233 (185/48)	200 (147/53)	Patients (P:189) vs. healthy individuals	Case–control study	SNaPshot system	7
Osman et al. (2016)	Saudi Arabia	**IL-1β:** rs16944, rs1143634 **IL-1α:** rs1800587 **TLR2:** rs3804099 **TLR4:** rs4986790, rs4986791 **IL-1R:** rs2234650 **TNF-α:** rs1800629, rs361525 **IL1RN:** rs315952	52 (22/30)	103 (44/59)	Hematogenous osteomyelitis patients vs. healthy individuals	Case–control study	PCR	6
Osman et al. (2015)	Saudi Arabia	**IL-4:** rs2070874, rs2243248 **IL-10:** rs1800896, rs1800871, rs1800872 **TGF-B1:** rs1800470 **IL-4R:** rs1801275 **IL-12B:** rs3212227 **IL-2:** rs2069762	52 (22/30)	103 (44/59)	Hematogenous osteomyelitis patients vs. healthy individuals	Case–control study	PCR	6
Kong et al. (2017)	China	**MMP1:** rs1799750, rs1144393	80 (41/40)	81 (37/44)	Osteomyelitis patients vs. healthy individuals	Case–control study	PCR-RFLP	6
Ocana et al. (2007)	Spain	**BAX:** G(-248)A	80 (53/27)	220	Osteomyelitis patients vs. healthy individuals	Case–control study	PCR-RFLP	8
Tsezou et al. (2008)	Greek	**IL-1α:** rs1800587 **IL-4:** rs2243248, rs2243250 **IL-6:** rs1800795	81 (42/39)	110 (62/48)	Osteomyelitis patients vs. healthy individuals	Case–control study	PCR-SSP assay	6

* *P/H/V represents the number of patients with posttraumatic osteomyelitis, or hematogenous osteomyelitis, or vascular insufficiency-related osteomyelitis*.

### Summary of the Outcomes

According to the included studies, we collected 25 SNPs in 17 significant genes (Table 2), and we classified the protein products encoded by the 17 significant genes into the following two categories.

**Table 2:** Summary of significant genes and polymorphisms of included studies.

**References**	**Gene**	**SNPs**	**Genotype**	Unadjusted *p*-value^*^	**OR (95%)**	**Risk or protective factor**
Jiang et al. (2020)	IL-1β	rs16944	GG+AG vs. AA	0.017	1.854 (0.988–1.741)	Risk factor
			GG vs. AA	0.041	1.831 (1.026–3.267)	Risk factor
			AG vs. AA	0.022	1.869 (1.093–3.194)	Risk factor
		rs1143627	TT+CT vs. CC	0.032	1.735 (1.050–2.868)	Risk factor
			TT vs. CC	0.040	1.839 (1.029–3.285)	Risk factor
Osman et al. (2016)	IL-1β	rs16944	GG vs. AG+AA	0.005	0.34 (0.16–0.73)	Protective factor
			AA vs. GG+GA	0.005	4.11 (1.51–11.21)	Risk factor
Asensi et al. (2003)	IL-1β	rs1143634	TT vs. CC+CT	0.014	6.98 (1.21–52.14)	Risk factor
Jiang et al. (2020)	IL1RN	rs4251961	C vs. T	0.01	0.519 (0.313–0.861)	Protective factor
			CC+CT vs. TT	0.005	0.446 (0.254–0.781)	Protective factor
			CT vs. TT	0.003	0.409 (0.227–0.737)	Protective factor
Jiang et al. (2020)	IL-6	rs1800796	CC+CG vs. GG	0.029	4.184 (1.154–15.165)	Risk factor
			CC vs. GG	0.026	4.378 (1.197–16.007)	Risk factor
			CG vs. GG	0.046	3.834 (1.024–14.347)	Risk factor
Tsezou et al. (2008)	IL-6:	rs1800795	CC+CG vs. GG	<0.01	2.05 (1.14–3.67)	Risk factor
Osman et al. (2015)	IL-4:	rs2070874	C vs. T	0.01	2.1 (1.92–3.71)	Risk factor
			CC vs. TT+TC	0.009	2.53 (1.25–5.13)	Risk factor
			CT vs. CC+TT	0.041	0.45 (0.21–0.97)	Protective factor
		rs2243248	GT vs. GG+TT	0.006	9.18 (1.87–45.0)	Risk factor
Tsezou et al. (2008)	IL-4:	rs2243248	TT+TG vs. GG	<0.01	21.33 (9.49–47.96)	Risk factor
		rs2243250	TT+TC vs. CC	<0.01	10.09 (5.15–19.75)	Risk factor
Osman et al. (2015)	IL-10	rs1800871	A vs. G	0.044	1.7 (1.01–2.98)	Risk factor
Osman et al. (2015)	IL-12B	rs3212227	GG vs. TT+TG	0.049	2.04 (1.0–4.16)	Risk factor
Tsezou et al. (2008)	IL-1α:	rs1800587	TT+TC vs. CC	<0.01	3.33 (1.78–6.24)	Risk factor
Asensi et al. (2003)	IL-1α	rs1800587	TT vs. CC+CT	0.008	3.7 (1.35–10.34)	Risk factor
Zhao et al. (2020)	IFN-γ	rs2430561	A vs. T	0.013	1.742 (1.122–2.705)	Risk factor
			AA + AT vs. TT	0.017	1.820 (1.111–2.983)	Risk factor
			AT vs. TT	0.029	1.781 (1.057–3.002)	Risk factor
Hou et al. (2018)	TNF-α	rs1799964	T vs. A	0.035	1.466 (1.025–2.095)	Risk factor
			TT vs. AA+AT	0.048	1.516 (1.004–2.290)	Risk factor
Osman et al. (2016)	TLR2	rs3804099	T vs. C	0.004	0.56 (0.34–0.94)	Protective factor
			TT vs. CC+CT	0.011	0.23 (0.08–0.71)	Protective factor
Montes et al. (2006)	TLR4	rs4986790	GG vs. AA+AG	0.038	NA	
		rs4986791	TT vs. CC+CT	0.038	NA	
Wang et al. (2017)	COX-2	rs689466	GG vs. AA+AG	0.018	1.74 (1.098–2.755)	Risk factor
Perez-Is et al. (2019)	CTSG	rs45567233	G vs. A	0.01	1.78 (1.14–2.78)	Risk factor
			AG vsGG+AA	0.015	1.78 (1.11–2.84)	Risk factor
Jiang et al. (2016)	VDR	rs731236	C vs. T	0.044	1.830 (1.009–3.319)	Risk factor
			CC+CT vs. TT	0.05	1.887 (1.001–3.319)	Risk factor
		rs2228570	C vs. T	0.029	1.347 (1.031–1.761)	Risk factor
			CC+CT vs. TT	0.042	1.594 (1.016–2.500)	Risk factor
			CC vs. TT	0.034	1.803 (1.046–3.106)	Risk factor
Kong et al. (2017)	MMP1	rs1144393	G vs. A	0.033	1.622 (1.038–2.536)	Risk factor
			GG vs. AA	0.024	2.792 (1.127–6.917)	Risk factor
		rs1799750	2G vs. 1G	0.014	1.735 (1.115–2.701)	Risk factor
			2G/2G vs. 1G/2G +1G/1G	0.025	2.605 (1.116–6.082)	Risk factor
Valle-Garay et al. (2013)	tPA	rs4646972	I vs. D	0.001	1.49 (1.17–1.91)	Risk factor
			I/I vs. I/D+D/D	<0.0001	2.59 (1.74–3.87)	Risk factor
Ocana et al. (2007)	BAX	G(-248)A	A vs. G	0.028	1.81 (1.06–3.07)	Risk factor

* *No special significance can be ascribed to associations with p < 0.05*.

#### Cytokine-Related Proteins

In seven case–control studies, 14 SNPs in nine genes encoding cytokine-related proteins (IL-1α, IL-1β, IL-1RN, IL-4, IL-6, IL-10, IL-12β, IFN-γ, and TNF-α) were investigated. Recently, Jiang et al. (2020) have reported a significant statistical difference in the genotype distribution of *IL1B* genes rs16944 and rs1143627 between patients with osteomyelitis and controls and revealed that the GG and AG genotypes of rs16944 and the TT and CT genotypes of rs1143627 were linked to the risk of posttraumatic osteomyelitis. Additionally, the CC and CG genotypes of rs1800796 located on the *IL6* gene were associated with an increased risk of posttraumatic osteomyelitis. However, the mutant C allele and CT genotype of rs4251961 within *IL1RN* were considered a protective factor against posttraumatic osteomyelitis. Additionally, Asensi et al. (2003) have revealed that the TT genotype within *IL1A* rs1800587 and *IL1B* rs1143634 might be associated with the occurrence of osteomyelitis. Among patients with osteomyelitis, the TT genotype in rs1800587 was significantly associated with a decreased age at which the diagnosis of osteomyelitis is made. Tsezou et al. (2008) have reported that the results of dominant genetic models used for rs1800587, rs2243248, rs2243250, and rs1800795 demonstrated that *IL1A, IL4*, and *IL6* SNPs could contribute to the genetic pathologies of osteomyelitis. The result of *IL1A* rs1800587 regarded as a risk factor for osteomyelitis was consistent with the outcome reported by Asensi et al. (2003). However, no significant difference was observed between patients with osteomyelitis and healthy controls regarding the individual alleles and genotypes of IL-1α rs1800587 in the studies of Jiang et al. (2020) and Osman et al. (2016). Osman et al. (2015) have reported that the population with the C allele or CC genotype might increase the susceptibility to hematogenous osteomyelitis, whereas the T allele or CT genotype could act as a protector factor. A heterozygous genetic model of rs2243248 has demonstrated that the mutant G allele contributed to hematogenous osteomyelitis, although the distribution difference of allele G and T frequencies among cases and controls was not statistically significant (*p* > 0.05). In addition, the allele A of rs1800871 within the *IL10* gene and the GG genotype of *IL12B* gene rs3212227 were identified to contribute to hematogenous osteomyelitis. Osman et al. (2016) have also revealed that the AA genotype of rs16944 in *IL1*β could be a risk factor, whereas the allele G and GG genotype of this SNP were considered a protector against hematogenous osteomyelitis among Saudis. However, this result was contrary to the conclusion of the study by Jiang et al. (2020). Zhao et al. (2020) have recently reported that the mutant allele A in rs2430561 might be a risk factor of posttraumatic osteomyelitis, and individuals with the AT genotype in this gene might have a higher risk of developing posttraumatic osteomyelitis. Hou et al. (2018) have first reported that individuals with the TT genotype of the rs1799964 SNP in *TNF* might have a higher risk of developing extremity chronical osteomyelitis in China. However, the results of a study (Asensi et al., 2003) indicated that rs1799964 in *TNF* is not associated with the susceptibility to the development of chronic bacterial osteomyelitis.

#### Protein, Receptor, and Enzyme

In another seven case–control studies, 11 SNPs in eight genes encoding a protein (BAX), receptors (VDR, TLR2, and TLR4), and enzymes (CTSG, COX-2, MMP-1, and t-PA) were investigated. Ocana et al. (2007) have found that the frequency of the mutant A allele at position 248 within the *BAX* gene was higher in patients with osteomyelitis than that in healthy controls, which was linked to the lower expression of BAX and prolonged survival of peripheral blood neutrophils. Jiang et al. (2016) have identified that the frequencies of the mutant C allele of rs731236 (*TaqI*) and rs2228570 (*FokI*) were higher in patients with chronic osteomyelitis than those in healthy individuals. The result of the dominant genetic model of rs731236 and the dominant and homozygous genetic models of rs2228570 has suggested that *VDR* SNPs are significantly linked to the susceptibility of developing chronic osteomyelitis. Different genotypes in rs731236 (*TaqI*) and rs2228570 (*FokI*) polymorphism were significantly associated with serum TNF-α levels in patients with osteomyelitis. Osman et al. (2016) have found that the mutant T allele and TT genotype of rs3804099 in the *TLR2* gene might protect against hematogenous osteomyelitis in the Saudi population. Montes et al. (2006) have demonstrated that although the contribution of the A allele of rs498670 and T allele of rs498671 in *TLR4* did not differ between patients and controls, the outcomes of the recessive genetic model of rs498670 (GG genotype) and rs498671 (TT genotype) revealed that individuals with GG or TT would have an increased susceptibility to osteomyelitis. Perez-Is et al. (2019) have reported that the G allele of rs45567233 situated in *CTSG* was more frequent in patients with osteomyelitis than that in controls. The result has suggested that the G allele could be a risk factor, and people with the AG genotype would elevate the risk of osteomyelitis in a Spanish population. The association of the rs45567233 polymorphism with the susceptibility of osteomyelitis might be achieved by elevating serum CTSG activity and lactoferrin levels. Wang et al. (2017) have concluded that the G allele and GG genotype of rs689466 located in the *PTGS2* gene (COX-2) could be considered a risk factor contributing to the onset of posttraumatic osteomyelitis. Serum C-reactive protein (*p* = 0.017) and IL-6 (*p* = 0.006) levels were significantly higher in patients with posttraumatic osteomyelitis accompanied with the GG genotype, instead of the CG genotype. Kong et al. (2017) have concluded that the G allele of rs1144393 in the MMP1 gene was regarded as a genetic risk factor and carriers of the GG genotype have an increased risk of osteomyelitis.

### Bioinformatics Analysis

**Figure 2:**
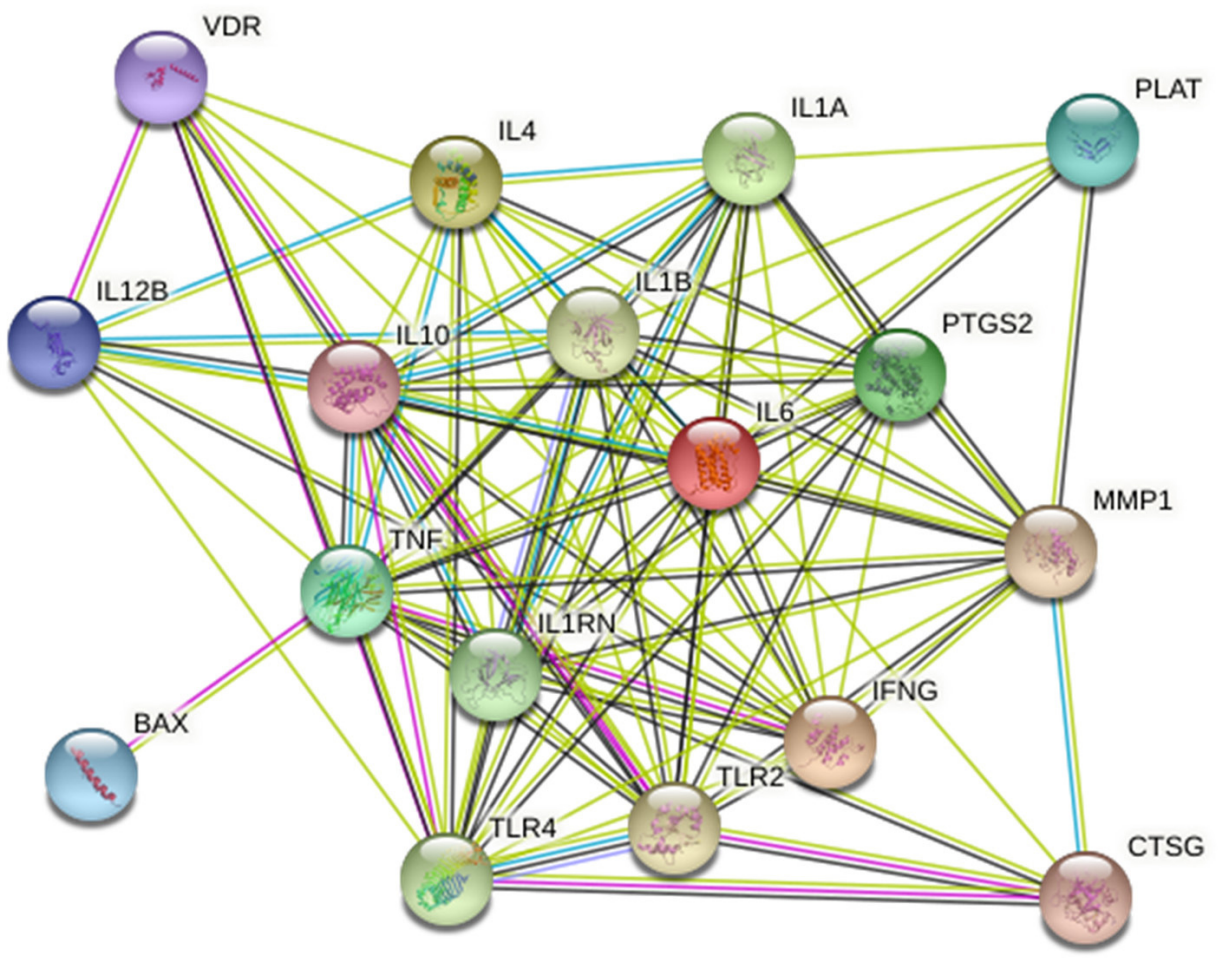
Protein–protein interaction network of osteomyelitis-related genes.

**Figure 3:**
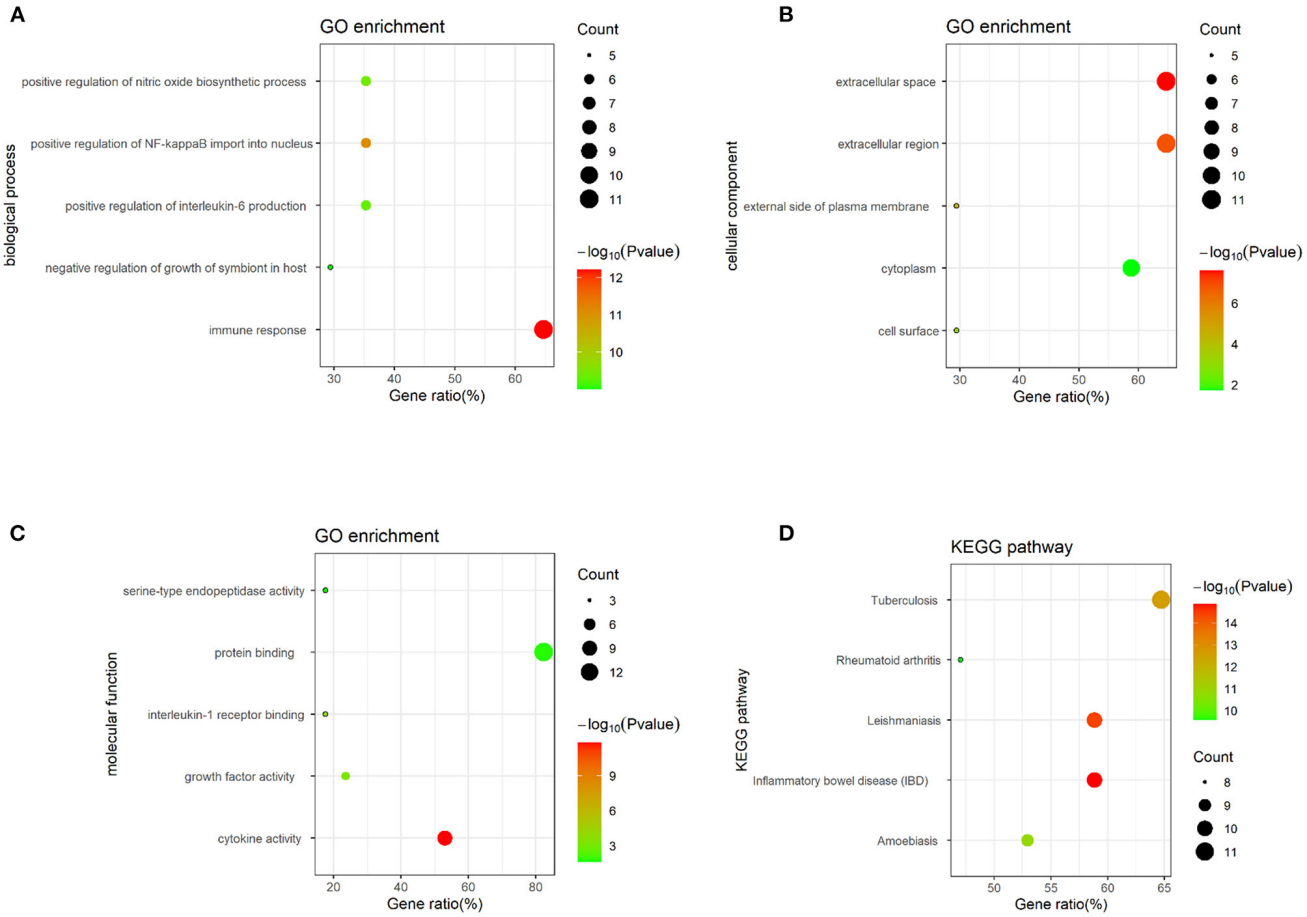
GO and KEGG analysis of osteomyelitis-related genes, containing **(A)** biological process, **(B)** cell component, **(C)** molecular function, and **(D)** biological pathway.

Twenty-five osteomyelitis-related genes were presented in the publications included in this review. The proteins encoded by osteomyelitis-related genes that were extracted from the included publications exhibited significant correlations (Figure 2). Among these genes, the node degree of TNF was the highest (Table 3). Additionally, the results of the GO and KEEG analysis of these osteomyelitis-related genes are shown in Figure 3. For biological processes, these genes were significantly enriched in the following terms: “immune response,” “positive regulation of NF-kappa B import into nucleus,” “positive regulation of nitric oxide biosynthetic process,” “positive regulation of interleukin-6 production,” and “negative regulation of growth of symbiont in host.” For cell components, these genes were enriched in the following terms: “extracellular space,” “extracellular region,” “external side of plasma membrane,” “cell surface,” and “cytoplasm.” For molecular functions, these genes were mainly enriched in the following terms: “cytokine activity,” “interleukin-1 receptor binding,” “growth factor activity,” “protein binding,” and “serine-type endopeptidase activity.” According to the result of the KEEG analysis, these genes mainly participated in the pathways including inflammatory bowel disease (IBD), leishmaniasis, tuberculosis, amoebiasis, and rheumatoid arthritis. The results indicated that inflammation is involved in the pathogenesis of osteomyelitis. In addition, 25 SNPs of 17 genes were reported to be significantly associated with the risk of osteomyelitis (Table 2). Many SNPs were mainly located in the intron or promotor region. According to regulome analysis, IL1B rs1143627, IL1B rs16944, IFNG rs2430561, and IL4 rs2070874 had scores of 1b, 1f, 2b, and 2b, respectively (Table 4). Smaller scores imply that SNPs have a greater biological functional significance.

**Table 3:** Hub genes among the PPI network.

**Gene symbol**	**Degree**	**Betweenness**
TNF	22	52.46152
TLR4	21	10.46152
CXCL8	21	10.46152
IL6	21	10.46152
TLR2	20	8.44535
IL17A	20	4.96152
IFNG	20	4.96152
IL4	20	4.96152
IL10	20	4.96152

**Table 4:** RegulomeDB score for all of these 23 selected SNPs.

**Chrom**	**Gene**	**SNP_ID**	**Functional consequence**	**Score ranking**	**Supporting data**
chr2	IL-1β	rs1143627	5′-UTR variant	1b	eQTL+ TF binding + any motif + DNase Footprint + DNase peak
chr2	IL-1β	rs16944	Upstream transcript variant	1f	eQTL+TF binding/DNase peak
chr12	IFN-γ	rs2430561	Intron variant	2b	TF binding + any motif + DNase Footprint + DNase peak
chr5	IL-4	rs2070874	5′-UTR variant	2b	TF binding + any motif + DNase Footprint + DNase peak
chr1	COX2	rs689466	Upstream variant	3a	TF binding + any motif + DNase peak
chr1	IL-10	rs1800871	Upstream transcript variant	3a	TF binding + any motif + DNase peak
chr6	TNF-α	rs1799964	Upstream transcript variant	3a	TF binding + any motif + DNase peak
chr11	MMP1	rs1144393	Upstream variant	4	TF binding + DNase peak
chr11	MMP1	rs1799750	Upstream variant	4	TF binding + DNase peak
chr12	VDR	rs731236	Synonymous variant	4	TF binding + DNase peak
chr14	CTSG	rs45567233	Missense variant	4	TF binding + DNase peak
chr2	IL1RN	rs4251961	Intron variant	4	TF binding + DNase peak
chr5	IL-4	rs2243248	Upstream transcript variant	4	TF binding + DNase peak
chr5	IL-4	rs2243250	Upstream transcript variant	4	TF binding + DNase peak
chr7	IL-6	rs1800796	5′-UTR variant	4	TF binding + DNase peak
chr7	IL-6	rs1800795	Intron variant	4	TF binding + DNase peak
chr12	VDR	rs2228570	Missense variant	5	TF binding or DNase peak
chr2	IL-1α	rs1800587	5′-UTR variant	5	TF binding or DNase peak
chr2	IL-1β	rs1143634	Synonymous variant	5	TF binding or DNase peak
chr9	TLR4	rs4986790	Missense variant	6	Other
chr4	TLR2	rs3804099	Synonymous variant	7	Other
chr5	IL12B	rs3212227	3′-UTR variant	7	Other
chr9	TLR4	rs4986791	Missense variant	7	Other

## Discussion

Bacterial osteomyelitis contains a complex inflammatory reaction caused by invading microorganisms. *S. aureus* is the bacterial pathogen frequently associated with posttraumatic and hematogenous osteomyelitis (Lew and Waldvogel, 2004). Despite appropriate treatments through medications and surgery, up to 30% of osteomyelitis cases become chronic, resulting in serious disability and economic burden (Lew and Waldvogel, 2004). To solve the problem of intractable chronic osteomyelitis disease, exploring the etiology and pathology of osteomyelitis is necessary.

The occurrence of bacterial osteomyelitis is a complicated process caused by both genetic and environmental factors. Much attention has been paid to exploring the close association between host factors in terms of gene polymorphisms and the risk of osteomyelitis. This review was conducted to summarize the SNPs of 25 genes linked to the susceptibility of osteomyelitis based on published literature. According to our review, rs1143627 and rs1143634 in *IL1B*, rs1800796 and rs1800795 in *IL6*, rs2243248 and rs2243250 in *IL4*, rs1800871 in *IL10*, rs3212227 in *IL12B*, rs1800587 in *IL1A*, rs2430561 in *IFNG*, rs1799964 in *TNF*, rs689466 in *PTGS2*, rs45567233 in *CTSG*, rs731236 and rs2228570 in *VDR*, rs1144393 and rs1799750 in *MMP1*, and rs4646972 in *PLAT*(t-PA) and *BAX*-248G/A increased the risk of osteomyelitis, whereas rs4251961 in *IL1RN*, rs380099 in *TLR2*, and the CT genotype of rs2070874 in *IL4* could protect against osteomyelitis. However, the results of Jiang et al. (2020) have suggested that the GG or GA genotype of *IL1B* rs16944 was a risk factor of osteomyelitis, which was inconsistent with the results of the study by Osman et al. (2016). The inconsistencies could be attributed to the insufficient sample size and population from different countries. Therefore, further research should be conducted to identify the link between *IL1B* gene polymorphism and osteomyelitis to clarify the etiology and pathogenesis of osteomyelitis.

Many genes collected from the publications included in this review can regulate the immune system and, therefore, contribute to host defense against pathogenic microorganisms in the bodily tissues and blood (Hill, 2012). Pathogen molecular patterns binding to pattern recognition receptors, such as Toll-like receptors (i.e., TLR2 and TLR4), can initiate inflammatory reactions of innate immune cells and induce the expression of pro-inflammatory cytokines (such as IL-1β and TNF-α). The activation of TLRs expressed in bone cells could influence osteoclast differentiation and activities in a complicated manner. TLRs expressed in early osteoclast precursors inhibit the differentiation of these cells, whereas the activation of TLR expressed in osteoblasts triggers the secretion of osteoclastogenic cytokines, including RANKL and TNF-α, which contribute to osteoclast differentiation and activation (Bar-Shavit, 2008). Yoshii et al. (2002) reported that pro-inflammatory IL-1β, IL-6, IL-4, and TNF-α levels in a locally infected bone increased during the infection period in a murine model of osteomyelitis, and IL-1β and IL-6 might contribute to bone damage during the earlier period of infection. IL-1R signaling contributes to bone destruction during osteomyelitis, whereas it also plays an important role in repressing local bacterial replication during bone infection (Putnam et al., 2019). IL-1β-activated osteoclasts exhibit strong absorbing ability and high H+ release (Shiratori et al., 2018). IL-10 is regarded as an immune modulatory cytokine that mitigates damage by decreasing the expression of inflammatory cytokines. IL-10 promoter polymorphisms seemed to be associated with the pathogenesis of chronic non-bacterial osteomyelitis (auto-inflammatory disorder) through the involvement of IL-10 dysfunction (Hofmann et al., 2011). Neutrophils are the first-line innate immune defense against many microbial infections. Meanwhile, the elimination of neutrophils through apoptosis or taken in by macrophages could alleviate the destructive nature of inflammation and promote resolution of the inflammation (Savill et al., 2002). BAX-α is a proapoptotic protein, and bcl-2 is an antiapoptotic protein (Oltvai et al., 1993). A high BAX-α/bcl-2 ratio would lead to apoptosis of leukemic cells (Pepper et al., 1998). *BAX* gene mutation could influence protein expression and biological function (Addeo et al., 2007). IFN-γ secreted by immunocytes in response to bacterial invasion strengthens antigen presentation and the phagocytic abilities of macrophages (Gomez et al., 2015). Meta-analyses of the association between IFN-γ+874T/A and susceptibility to leukemia (Wu et al., 2016), hepatocellular carcinoma (Zhou et al., 2015), and asthma (Nie et al., 2014) have been conducted. Matrix metalloproteinases (MMPs), a family of enzymes, play an important role in the degradation and rebuild of extracellular matrix under both normal physiological and pathological conditions. MMPs are involved in matrix degradation and joint destruction in arthritis diseases (Pap et al., 2000; Tetlow et al., 2001). The expression of inducible MMPs is increased by stimulating inflammatory mediators, such as TNF-α and IL-1 (Nagase and Woessner, 1999). A population-based study has discovered that the MMP1–1607(1G/2G) polymorphism might be associated with reduced bone mineral density at the distal radius in postmenopausal women (Yamada et al., 2002). Cathepsin G (CTSG) is a serine protease stored in the neutrophil azurophilic granules and has antimicrobial properties (Miyasaki et al., 1995). In addition, CTSG could activate extracellular MMPs at the site of inflammation, causing the degradation of extracellular matrix components (Baggiolini et al., 1978; Korkmaz et al., 2008). CTSG also activates osteoclast precursors by stimulating the expression of RANKL, enhancing mammary tumor-induced osteolysis (Beaujouin and Liaudet-Coopman, 2008). Vitamin D is essential in calcium homeostasis and bone metabolism. In addition, it participates in the regulation of inflammatory reactions (Yin and Agrawal, 2014). VDR is coded by the *VDR* gene located on chromosome 12. In addition, *VDR* gene polymorphisms affect the biological function of VDR. The gene polymorphisms *TaqI, BsmI, FokI*, and *ApaI* were most frequently investigated in many skeletal diseases. A case–control study has reported that the VDR *FokI* polymorphism was linked to the risk of osteoporosis in postmenopausal women (Wu et al., 2019). Recently, the results of a meta-analysis have suggested that VDR *BsmI* and *TaqI* polymorphisms were associated with the susceptibility to osteoarthritis in the spine (Cezar-Dos-Santos et al., 2020). Simultaneously, our bioinformatics analysis discovered that these 25 genes were associated with immune responses (biological process), extracellular space (cellular component), and cytokine activity (molecular function). Interestedly, these osteomyelitis-related genes were enriched in the disease pathways, including IBD. Inflammation participates in the pathogenesis of bacterial osteomyelitis from the aspect of bioinformatics analysis.

Four SNPs (*IL1B* rs1143627, *IL1B* rs16944, *IFNG* rs2430561, and *IL4* rs2070874) had smaller scores from the regulome analysis, implying that these SNPs had significant biological functions. rs1143627 and rs2070874 were located in the 5′-UTR region of *IL1B* and *IL4* gene, respectively. rs16944 is located in an intron of the *IL1B* gene, and rs2430561 is located in the *IFNG* promoter region. Thus, we can hypothesize that these SNPs contribute to the risk of osteomyelitis, possibly by affecting transcription factors or other molecules binding to the motif.

This review summarized a series of genes and SNPs associated with osteomyelitis. Besides, the emergence of genome-wide association studies (GWAS) provides many opportunities to identify alleles associated with complex diseases (Altshuler et al., 2008). A GWAS from the UK Biobank (http://geneatlas.roslin.ed.ac.uk/; Bycroft et al., 2018) involved 452,264 individuals, including 698 patients with osteomyelitis. The subjects were from the UK and were between 40 and 69 years old. SNP genotyping was identified using the UK Biobank AXIOM array. However, unfortunately, we have not found any published data about the relationship between several SNPs and the risk of osteomyelitis using GWAS.

Genetic variants exist among individuals, but the influence of these genetic polymorphisms on clinical significance or phenotypic diversity has not been known yet. Lappalainen et al. (2013) have demonstrated that genetic variation could affect the occurrence and development of diseases by regulating gene expression. Thus, elucidating how genotype varieties clinically affect phenotypes in complicated diseases remains challenging. Exploring the role of genetics in phenotypes and diseases and its potential interactions with other factors is of great significance for understanding pathogenesis of diseases. It is hoped that this will provide more opportunities for drug development and personalized treatments. Note that human genetics is a valuable tool for the therapeutic hypothesis in drug development. Plenge et al. (2013) have provided empirical examples of drug–gene pairs and objective criteria to highlight the role of genetic findings for drug discovery in the future. For example, anakinra (interleukin-1 receptor antagonist) treatment is effective in patients with adult-onset Still disease in the preliminary experience (Lequerre et al., 2008). Similarly, according to our summary of genetic SNPs associated with bacterial osteomyelitis, some inflammatory cytokine-related inhibitors can potentially be used to test therapeutic hypotheses for the development of drugs and treatment of bacterial osteomyelitis, such as ustekinumab (anti-IL12 monoclonal antibody), tocilizumab (anti-IL6R monoclonal antibody), and anakinra (interleukin-1 receptor antagonist).

In this review, we summarized the association of gene polymorphisms with the susceptibility to osteomyelitis. However, this review has many limitations as well. The primary limitations of this review include the following: (1) the limited number of available studies and individuals selected for this review; (2) the low statistical efficacy caused by the limited sample size; (3) the limitation of ethnic diversity; (4) three different types of osteomyelitis that were not analyzed separately; and (5) the limited amount of data extracted from few studies, preventing us from conducting more quantitative analysis (meta-analysis).

## Conclusion

This review summarized the association between gene polymorphisms and the increasing risk of osteomyelitis. According to this review, rs1143627 and rs1143634 in *IL-1B*, rs1800796 and rs1800795 in *IL6*, rs2243248 and rs2243250 in *IL4*, rs1800871 in *IL10*, rs3212227 in *IL12B*, rs1800587 in *IL1A*, rs2430561 in *IFNG*, rs1799964 in *TNF*, rs689466 in *PTGS2*, rs45567233 in *CTSG*, rs731236 and rs2228570 in *VDR*, rs1144393 and rs1799750 in *MMP1*, and rs4646972 in *PLAT*(t-PA) and *BAX*-248G/A increased the risk of osteomyelitis, whereas rs4251961 in *IL1RN*, rs380099 in *TLR2*, and the CT genotype, not the CC genotype, of rs2070874 in *IL4* could protect against osteomyelitis. However, due to the small sample size included in this review, we cannot draw a definitive conclusion on the correlation between genetic polymorphisms and susceptibility to osteomyelitis. Therefore, large-scale prospective studies should be conducted to further illustrate the relationship between SNPs and the risk of developing osteomyelitis. Meanwhile, investigating how genetic diversity influences clinical phenotypes is also necessary to better understand the role of genetic factors in the pathogenesis of osteomyelitis and to provide more personalized preventions and interventions for osteomyelitis in clinical practice.

## Data Availability Statement

The original contributions presented in the study are included in the article/Supplementary Material, further inquiries can be directed to the corresponding author/s.

## Author Contributions

XX and JL designed the study and collected the data. XX drafted the manuscript. FG, KZ, ZSu, QW, ZSui, and PZ contributed to the writing. TY provided critical feedback and contributed to the review of the manuscript. All authors contributed to the article and approved the submitted version.

## Conflict of Interest

The authors declare that the research was conducted in the absence of any commercial or financial relationships that could be construed as a potential conflict of interest.
