# Supplementary material for: Genetic Determinants for Bacterial Osteomyelitis: A Focused Systematic Review of Published Literature
Source: Front Genet. 2021 Jun 17;12:654792. doi: 10.3389/fgene.2021.654792 (PMC8248359; doi:10.3389/fgene.2021.654792)
Supplement: Supplementary file 1 [file Table_1.DOCX]

**Supplementary Table 1. Summary of the search strategies.**

| **PUBMED** | | |
| --- | --- | --- |
| Search | Query | Results |
| #1 | ("Polymorphism, Genetic"[Mesh] ) OR ("Polymorphism, Single Nucleotide"[Mesh]) OR (genetic variants) OR (DNA polymorphism) OR (SNP) | 507,269 |
| #2 | (osteomyelitis[Mesh Terms]) OR (bone infection) | 133,311 |
| #3 | #1 AND #2 | 999 |
|  |  |  |
| **Embase** | | |
| #1 | ‘genetic polymorphism’ OR ‘dna polymorphism’ OR ‘single nucleotide polymorphism’ | 378,180 |
| #2 | osteomyelitis' OR 'bone infection' | 47,761 |
| #3 | #1 AND #2 | 119 |
|  | | |
| **Web of Science** | | |
| #1 | TS=(osteomyelitis OR bone infection) | 251,791 |
| #2 | TS=(genetic polymorphism OR genetic variant OR DNA polymorphism OR single nucleotide polymorphism OR SNP | 657,859 |
| #3 | #1 AND #2 | 2,787 |
